# The pathway of impacts of aerosol direct effects on secondary inorganic aerosol formation

**DOI:** 10.5194/acp-22-5147-2022

**Published:** 2022-04-20

**Authors:** Jiandong Wang, Jia Xing, Shuxiao Wang, Rohit Mathur, Jiaping Wang, Yuqiang Zhang, Chao Liu, Jonathan Pleim, Dian Ding, Xing Chang, Jingkun Jiang, Peng Zhao, Shovan Kumar Sahu, Yuzhi Jin, David C. Wong, Jiming Hao

**Affiliations:** 1 Key Laboratory of Aerosol and Cloud Precipitation of China Meteorological Administration, School of Atmospheric Physics, Nanjing University of Information Science and Technology, Nanjing, 210044, China; 2 State Key Joint Laboratory of Environmental Simulation and Pollution Control, School of Environment, Tsinghua University, Beijing, 100084, China; 3 U.S. Environmental Protection Agency, Research Triangle Park, NC 27711, USA; 4 Jiangsu Provincial Collaborative Innovation Center for Climate Change, School of Atmospheric Sciences, Nanjing University, Nanjing, 210023, China; 5 Nicholas School of the Environment, Duke University, Durham, NC 27710, USA; 6 Department of Health and Environmental Sciences, Xi’an Jiaotong-Liverpool University, Suzhou, 215123, China

## Abstract

Airborne aerosols reduce surface solar radiation through light scattering and absorption (aerosol direct effects, ADEs), influence regional meteorology, and further affect atmospheric chemical reactions and aerosol concentrations. The inhibition of turbulence and the strengthened atmospheric stability induced by ADEs increases surface primary aerosol concentration, but the pathway of ADE impacts on secondary aerosol is still unclear. In this study, the online coupled meteorological and chemistry model (WRF–CMAQ; Weather Research and Forecasting–Community Multiscale Air Quality) with integrated process analysis was applied to explore how ADEs affect secondary aerosol formation through changes in atmospheric dynamics and photolysis processes. The meteorological condition and air quality in the Jing-Jin-Ji area (denoted JJJ, including Beijing, Tianjin, and Hebei Province in China) in January and July 2013 were simulated to represent winter and summer conditions, respectively. Our results show that ADEs through the photolysis pathway inhibit sulfate formation during winter in the JJJ region and promote sulfate formation in July. The differences are attributed to the alteration of effective actinic flux affected by single-scattering albedo (SSA). ADEs through the dynamics pathway act as an equally or even more important route compared with the photolysis pathway in affecting secondary aerosol concentration in both summer and winter. ADEs through dynamics traps formed sulfate within the planetary boundary layer (PBL) which increases sulfate concentration in winter. Meanwhile, the impact of ADEs through dynamics is mainly reflected in the increase of gaseous-precursor concentrations within the PBL which enhances secondary aerosol formation in summer. For nitrate, reduced upward transport of precursors restrains the formation at high altitude and eventually lowers the nitrate concentration within the PBL in winter, while such weakened vertical transport of precursors increases nitrate concentration within the PBL in summer, since nitrate is mainly formed near the surface ground.

## 1 Introduction

Aerosols have long been recognized as a major source of uncertainty in the climate system (Carslaw et al., 2013; Koch and Del Genio, 2010; Ramanathan et al., 2001; Rosenfeld et al., 2014). They perturb Earth’s energy budget through aerosol direct effects (ADEs) by direct scattering and absorbing shortwave and longwave radiation and indirect effects via interaction with clouds. Besides the climatic effects, studies in recent decades have revealed that it alters regional weather (Sun and Zhao, 2021; Zhao et al., 2018, 2020). Airborne aerosols can alter planetary boundary layer (PBL) development (Atwater, 1971; Ackerman, 1977; Ramanathan et al., 2001; Wendisch et al., 2008; Grell et al., 2011; Wong et al., 2012; Barbaro et al., 2013; Wang et al., 2013) and further deteriorate air quality, which is defined as aerosol–PBL interactions (Ding et al., 2013; Wang et al., 2014; Xing et al., 2015a, 2016; Z. Wang et al., 2018; Huang et al., 2016; Wang et al., 2015; Yang et al., 2016a; Hong et al., 2020). Absorption and scattering of aerosols reduce the solar radiation reaching to the ground which lowers the surface temperature (McCormick and Ludwig, 1967; Li et al., 2016; Yang et al., 2016b, 2018). Meanwhile, aerosols can heat up the air in the upper layer with the presence of absorbing components (black carbon, brown carbon, and dust) (Ding et al., 2016; Huang et al., 2018; H. Wang et al., 2018). Such controversial effects modify the vertical temperature profile and suppress the development of the PBL, resulting in the accumulation of pollutants in the near-surface layer and aggravation of atmospheric pollution (Huang and Ding, 2021).

Compared to the impact pathways of ADEs on primary aerosol through the inhibition of PBL development, ADE effects on secondary aerosol, which is formed in the atmosphere through atmospheric reaction, are much more complicated. ADEs can affect secondary aerosol by changing vertical/horizontal transport and altering its precursors and reaction rate (Li et al., 2017; Liao et al., 2015; Ding et al., 2016; Yang et al., 2017). Studies have been conducted to explain the impact of aerosol on atmospheric oxidations through attenuation. He and Carmichael (1999) illustrated the distinct roles of different types of aerosols on the photochemical reaction rate and ozone (O_3_) concentration. Atmospheric aerosols cause significant attenuation of ultraviolet radiation and affect photolysis rates and species chemical cycles (Deng et al., 2012; Mok et al., 2016). Zheng et al. (2015) showed that oxidant concentrations fall dramatically during high aerosol loading in winter, suggesting a reduction in secondary aerosols through gaseous reactions. However, impacts of ADEs on secondary particle formation through atmospheric dynamic processes have not been well studied. Reduced ventilation by ADEs will concentrate gaseous precursors, thereby changing secondary particle formation in the surface and upper layers and indirectly influencing the aerosol concentration. Additionally, since secondary aerosol could either form in upper layers and get transported to near ground level or form at near ground level and get transported aloft, the modulation of PBL development due to ADEs may either increase or decrease surface-level secondary aerosol concentrations. A detailed understanding of the physical processes causing these impacts on near-surface and free-tropospheric aerosol burden and their quantification is still needed as is the relative importance of each pathway and their likely seasonal variation. To gain further insight into these pathways, process analysis is conducted in this study.

With the rapid development of the economy and the acceleration of urbanization, air quality in China has been deteriorating in recent decades, and extreme air pollution events have occurred frequently across China (H. Wang et al., 2018). In 2010, the population-weighted PM_2.5_ concentration in China was as high as 59 μg m^−3^. More than 80 % of the residents live in regions where 5-year-averaged PM_2.5_ is above the national Class II regional air quality standards (i.e., more than 35 μg m^−3^) (Apte et al., 2015). In 2013, annual-averaged PM_2.5_ concentrations across 74 key cities in China ranged from 26 to 160 μg m^−3^, with many locations far exceeding China’s air quality standard. The number of premature deaths due to exposure to PM_2.5_ in China is estimated to be more than 1 million for 2010 conditions (Wang et al., 2017; Lim et al., 2012; Apte et al., 2015). The air quality in China has improved significantly since 2013, owing to the strict control acts in China (Fan et al., 2020; Zhang et al., 2020). But understanding the causes of heavy-pollution incidents is needed for developing effective pollution control measures in China. To provide an insight into these questions, this study analyzes the contribution of each pathway for secondary inorganic aerosols. The diurnal and seasonal variations in these pathways are also explored. Investigation on the influence of ADEs on atmospheric pollution will provide important guidance for understanding the cause of atmospheric pollution and developing effective control strategies.

## 2 Methods

The overall modeling methodology for the study is detailed previously in Xing et al. (2017) and is briefly summarized here. In this study, the two-way coupled WRF–CMAQ meteorology–chemistry transport model (Weather Research and Forecasting–Community Multiscale Air Quality; Wong et al., 2012) was used to simulate the ADE impacts. Meteorology was simulated by the Weather Research and Forecasting (WRF) model version 3.4 developed by the National Center for Atmospheric Research (NCAR). Meteorological input data were the NCEP–NCAR Reanalysis (National Centers for Environmental Prediction) data. The Pleim–Xiu land surface model (Pleim and Xiu, 2003; Pleim and Gilliam, 2009), associated with version 2 of the Asymmetric Convective Model (ACM2) PBL scheme was used in this study. The MODIS land-use type was chosen. The RRTMG (Rapid Radiative Transfer Model for GCMs; general circulation models) radiation parameterization scheme was used for shortwave and longwave radiation treatment. The Morrison double-moment microphysics scheme and Kain–Fritsch cumulus scheme were used in this study. NCEP Automated Data Processing (ADP) global surface and upper-air observation data were carried out for four-dimensional data assimilation (grid FDDA). The air quality model used in this study was the Community Multiscale Air Quality Modeling System (CMAQ) version 5.0.1, developed by the Environmental Protection Agency of the United States (US EPA Office of Research and Development, 2012). In our previous papers, we have detailed and fully evaluated the model (Xing et al., 2015a, b; Wang et al., 2014; Xing et al., 2017). The comparison of simulated and observed PM_2.5_ concentration is shown in Fig. S1 in the Supplement. Gaseous species and aerosols were simulated by using the Carbon Bond 05 (CB05) gas-phase chemistry (Sarwar et al., 2008) with the AERO6 aerosol module (Appel et al., 2013). The BHCOAT coated-sphere module (Bohren and Huffman, 1983) was used to simulate aerosol optical properties based on simulated aerosol composition and size distribution (Gan et al., 2015). The gridded emission inventory and initial and boundary conditions used in this study were consistent with our previous studies (Wang et al., 2011; Zhao et al., 2013b, a; Wang et al., 2014).

**Figure 1.**
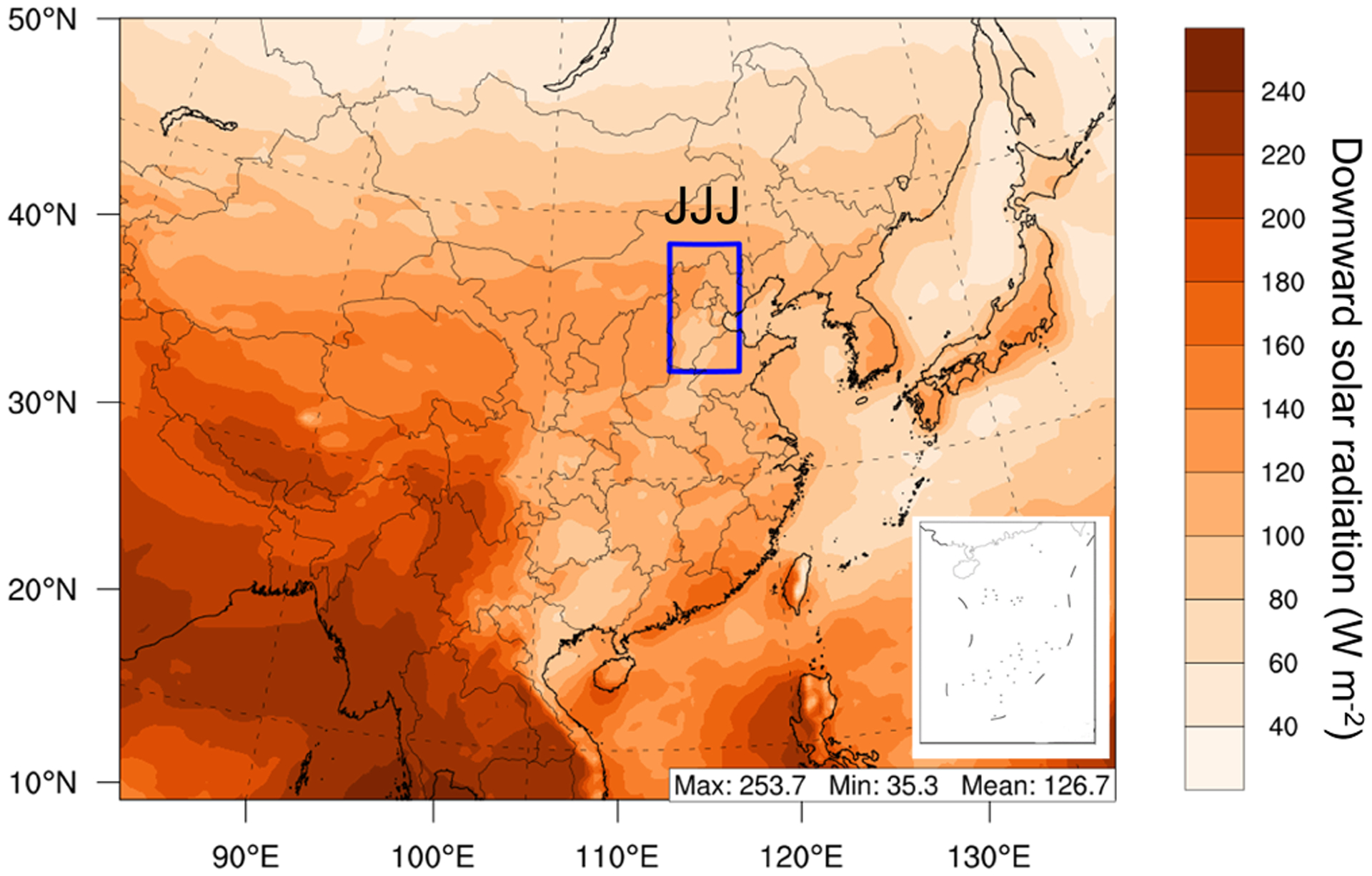
Simulation domain and locations of the Jing-Jin-Ji region in China. The color shows simulated daily average downward shortwave solar radiation (SWDOWN) at the bottom in January 2013.

Figure 1 shows the modeling domain, which covers most of China and surrounding portions of East Asia, discretized with a 36 km × 36 km grid resolution. WRF and CMAQ both use 23 vertical layers, in which 8 layers are set under 1 km to better describe the boundary layer processes; 1 to 31 January and 1 to 31 July in 2013 were selected to represent winter and summer conditions, respectively. Each simulation was also preceded by a 7 d spin-up period. The Jing-Jin-Ji area (denoted JJJ), including Beijing, Tianjin, and Hebei Province in China, was selected for the analysis. In this study, observation data from the China National Urban Air Quality Real-time Publishing Platform supported by the Ministry of Ecology and Environment, China, were used to evaluate the model performance. The validation results were shown as Figs. S1 to S4 in the Supplement.

Following our previous analyses (Xing et al., 2017), three scenarios were simulated, including (1) the baseline simulation (denoted SimBL) in which no aerosol photolysis rate changes or dynamics were considered, (2) the simulation (denoted SimNF) in which aerosol only affects photolysis rates, and (3) the simulation (denoted SimSF) in which aerosol feedbacks were considered through both photolysis and dynamic processes. The differences between the simulations of SimNF and SimBL were used to present the ADE impacts through the photochemistry process (ADEP, denoted photolysis in the figures). Similarly, the differences between the simulations of SimSF and SimNF were used to estimate the ADE impacts through the dynamic process (ADED, denoted dynamics in the figures). The combined ADE impacts due to both photolysis and dynamics (denoted Δ total) were estimated from the differences between the simulations of SimSF and SimBL.

To further explore these impacts, process analysis (PA) technology (Gipson and Young, 1999) was applied in the simulation of WRF–CMAQ (Xing et al., 2011). An Eulerian chemistry transport model simulates air pollution concentration by solving transport partial differential equations. A series of physical and chemical processes is calculated to determine the changes in species concentration at each time step. Based on the properties of the linear equation, process analysis could estimate the accumulated effects of each process. The integrated process rates (IPRs) quantify the hourly tendencies from six major modeled atmospheric processes shaping the simulated aerosol concentrations. These process tendencies represent the dominant sinks or sources and include aerosol process (denoted AERO), cloud processes (i.e., among others, the net effect of cloud attenuation of photolytic rates, denoted CLDS, and aqueous-phase chemistry), emission (denoted EMIS), dry deposition (denoted DDEP), horizontal advection (denoted HADV), horizontal diffusion (denoted HDIF), vertical advection (denoted ZADV), and vertical diffusion (denoted VDIF). We combined VDIF, ZADV, and DDEP into vertical transport (VTRN) and combined HDIF and HADV into horizontal transport (HTRN).

## 3 Results and discussion

**Figure 2.**
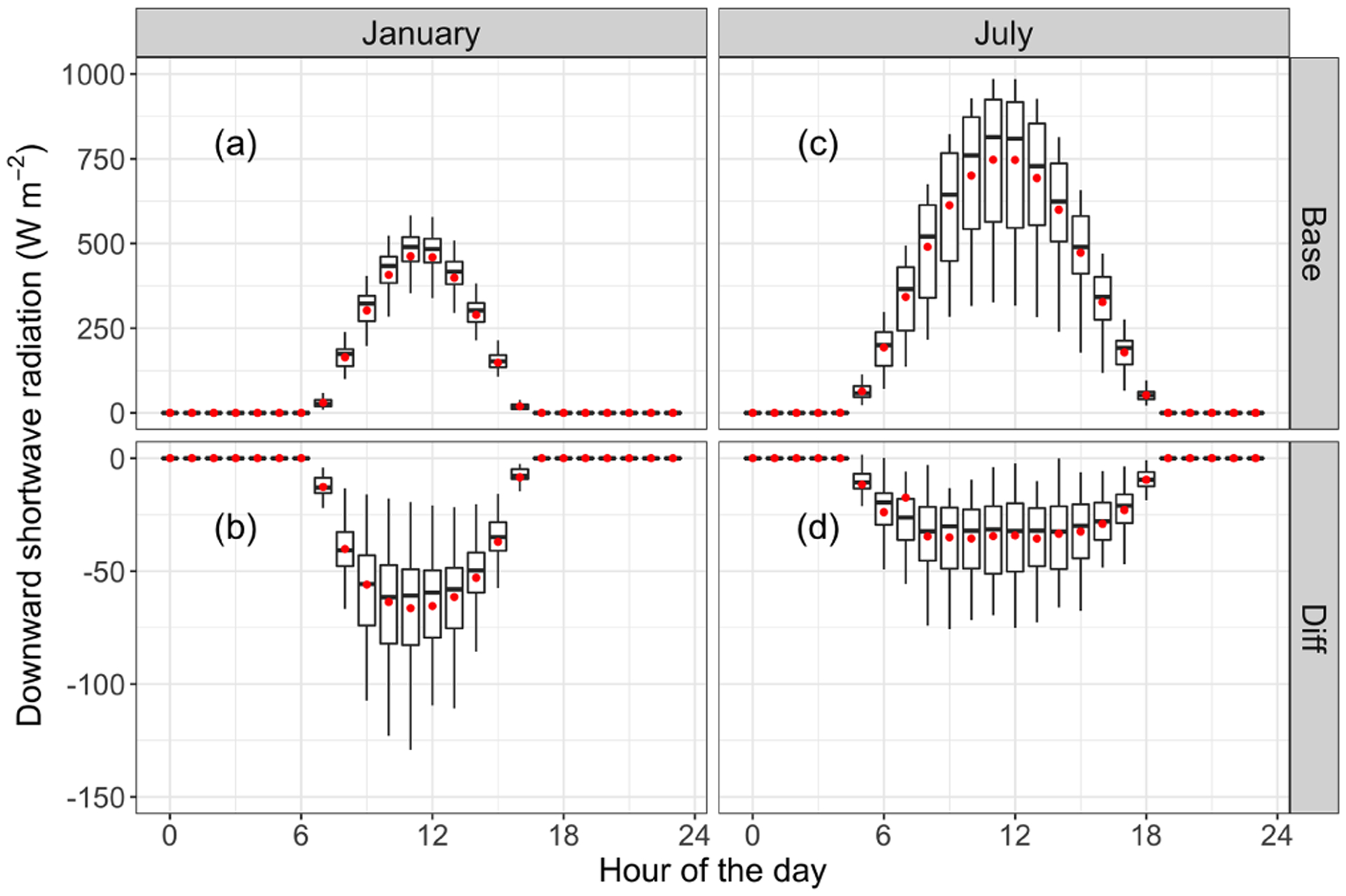
Diurnal variances of SWDOWN **(a, c)** and the impact of ADEs on SWDOWN **(b, d)**, in January and July 2013. The central rectangle spans the first quartile to the third quartile. The segment and red dot inside the rectangle show the median and mean value, respectively. The whiskers above and below the box extend to the highest and lowest values.

**Figure 3.**
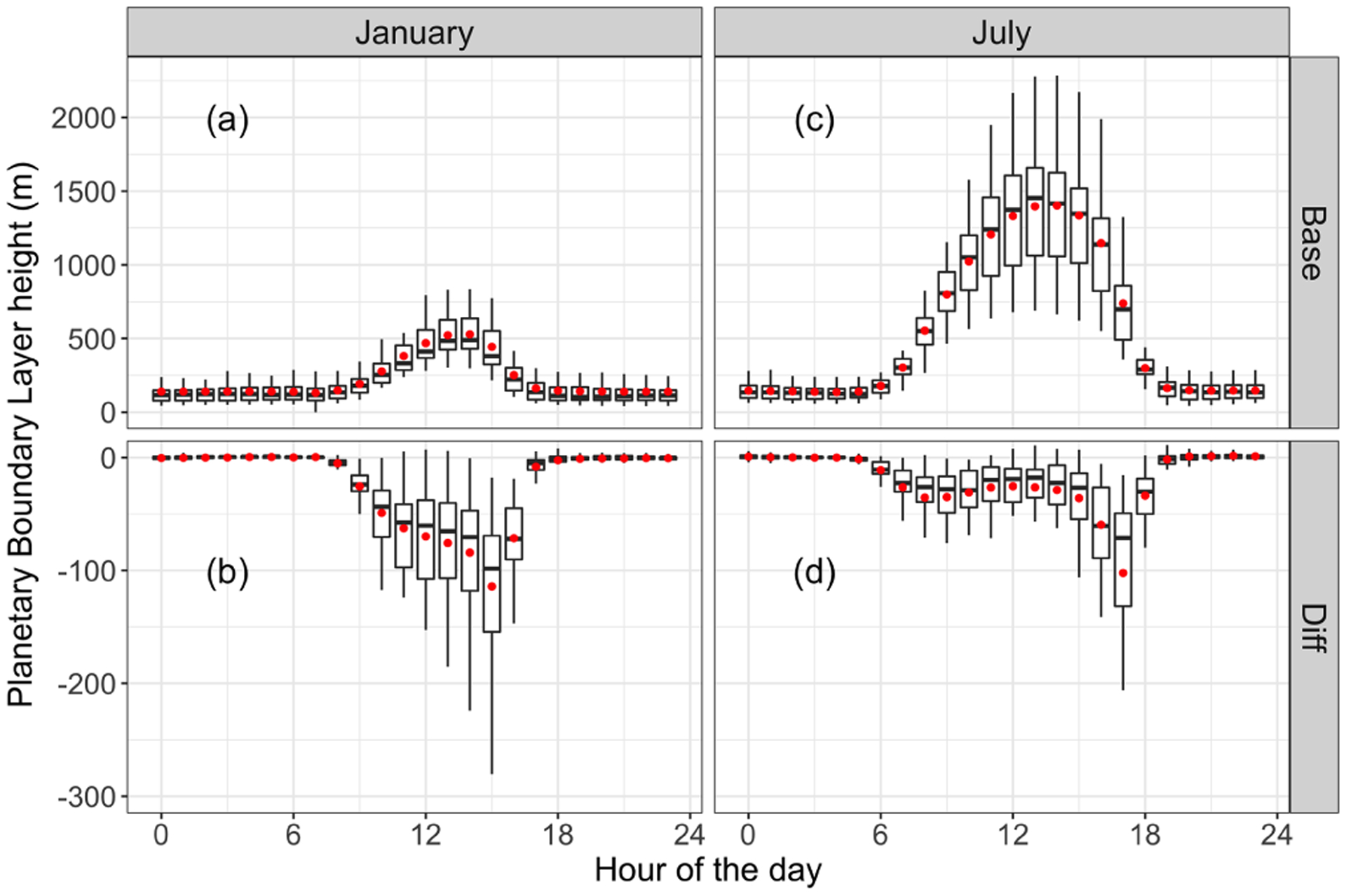
Diurnal variances of planetary boundary layer (PBL) height **(a, c)** and the impact of ADEs on PBL height **(b, d)**, in January and July 2013. The central rectangle spans the first quartile to the third quartile. The segment and red dot inside the rectangle show the median and mean value, respectively. The whiskers above and below the box extend to the highest and lowest values.

The perturbation of ADEs on solar radiation and the PBL is presented in Figs. 2 and 3, respectively. As shown in Fig. 2, ADEs reduce solar radiation reaching the ground. The daily maximum reduction occurs at noon, with a mean value of 70 and 40 W m^−2^ in January and July, respectively. Decreased solar radiation weakens surface turbulence and reduces the daily maximum PBL height. Figure 3 illustrates that the impact of ADEs on monthly mean PBL height shows a uni-modal distribution in January and bimodal distribution in July. The PBL height is reduced mostly in the afternoon. The daily average reduction in January and July is about 70 and 30 m, respectively. Meanwhile, the daily maximum PBL heights are about 500 and 1500 m in January and July, respectively. It indicates that the change of PBL height is more significant in January.

**Figure 4.**
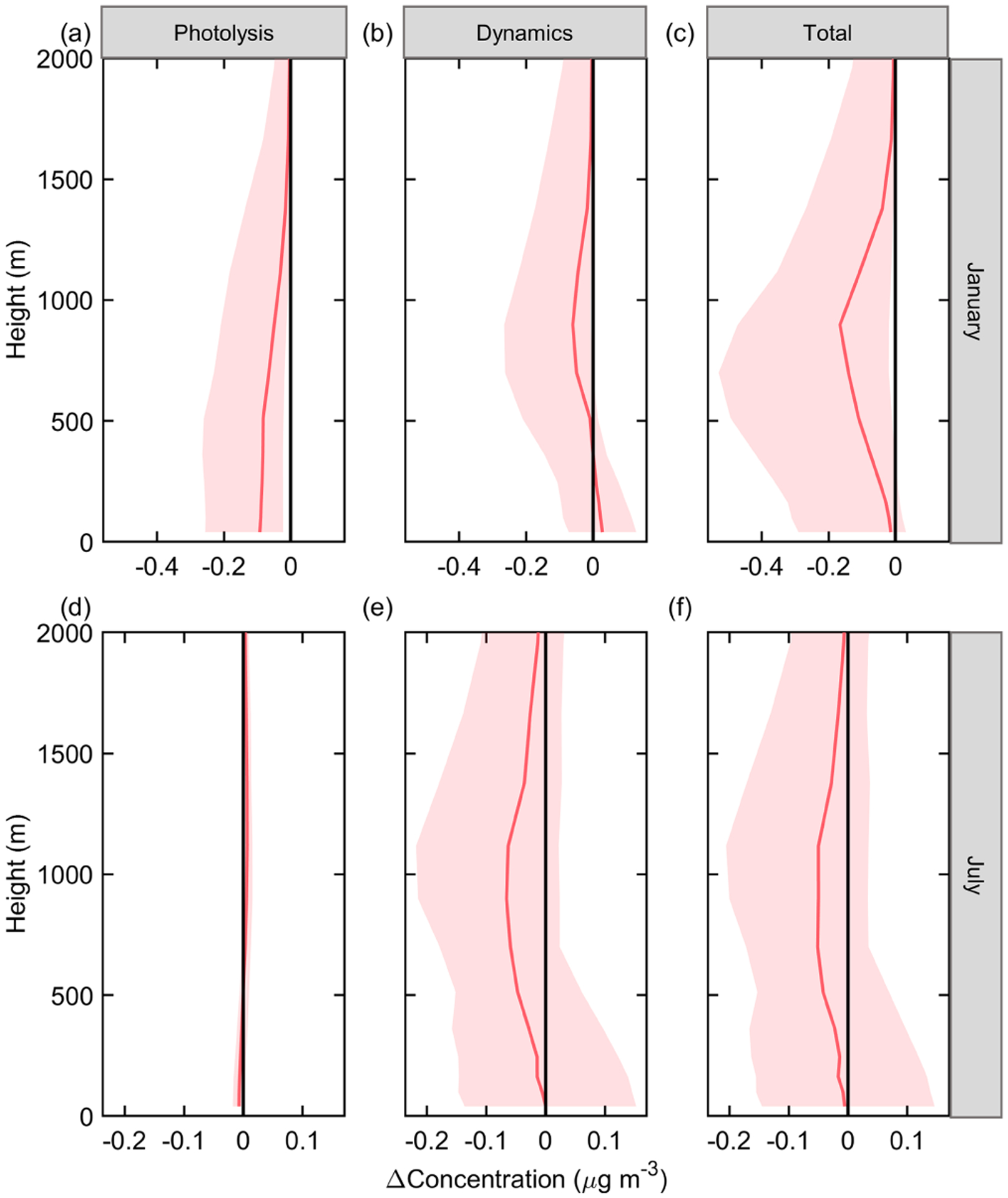
Vertical profile of sulfate concentration change to ADEs in the JJJ region at noontime in January **(a, b, c)** and July **(d, e, f)**.

**Figure 5.**
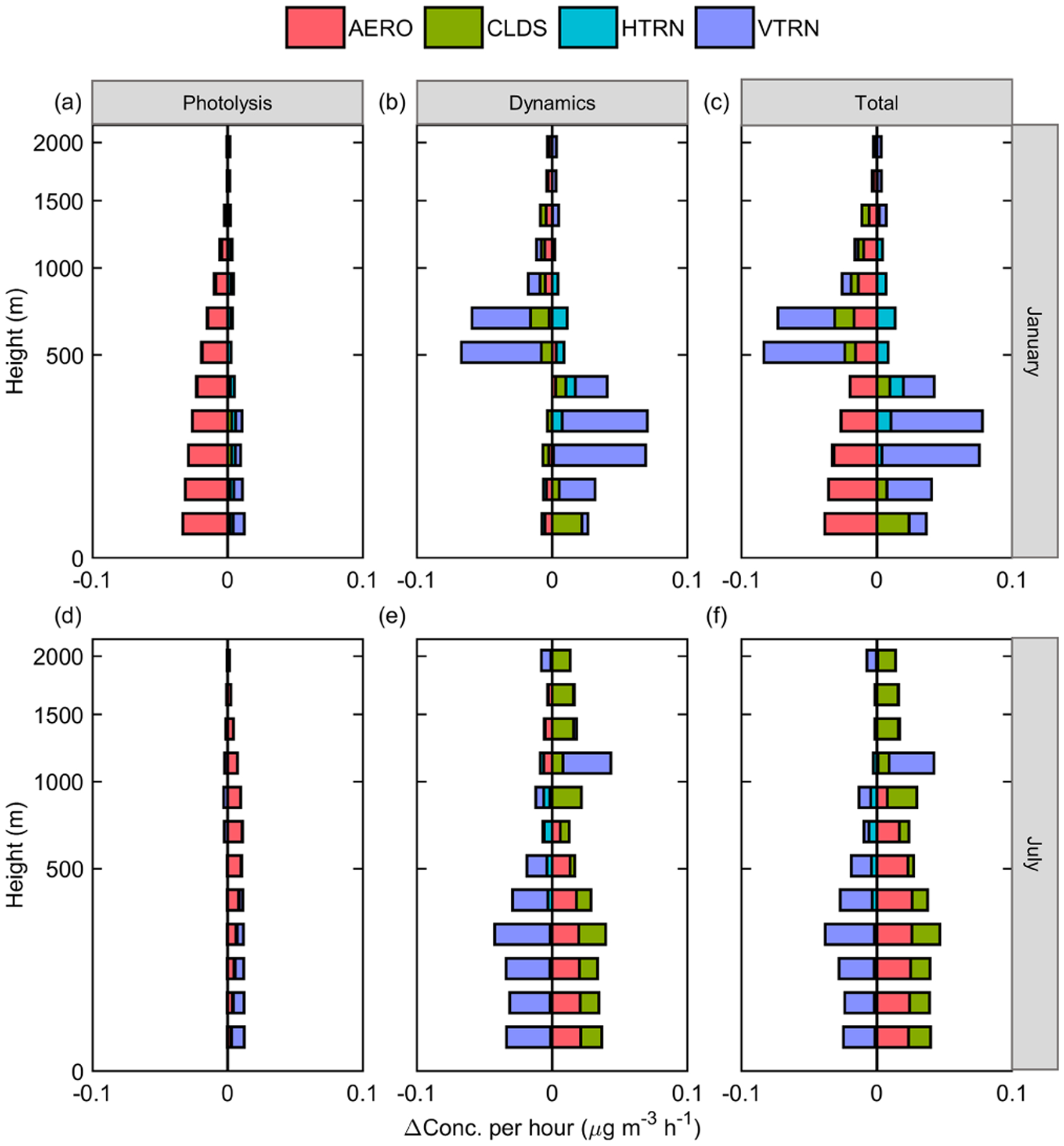
Vertical distribution of the responses of the main process of sulfate to ADEs in the JJJ region at noontime in January **(a, b, c)** and July **(d, e, f)**.

To provide insight into how ADEs affect sulfate concentration, the vertical distribution of sulfate concentration and related process responses to ADEs is presented in Figs. 4 and 5. As shown in Fig. 4, ADEs affect sulfate through both photolysis and dynamics in January, leading to a decrease of sulfate formation rate in all layers. The reduction rate due to ADEP is about 3 % on average in the near-surface layer. Dynamic processes lead to an increase in sulfate concentration in the near-surface layer and a decrease of sulfate concentration above 300 m. These two processes combined contribute a 7.5 % reduction of sulfate at 900 m, which is the strongest affected layer in terms of sulfate concentration. In July, the ADED is the key process altering sulfate concentration. The strongest impact is at 1100 m. Traditionally, the pathway through the changing of actinic flux is emphasized (Liao et al., 1999), but the pathway through the dynamic process and the further change of gaseous precursors are barely mentioned. Our results indicate that ADEs affecting sulfate formation through the dynamic pathway are equally or even more important than that of photolysis pathway in both summer and winter.

The vertical distribution of the sulfate IPRs response to ADEs is presented in Fig. 5. The vertical profile at noon is chosen for discussion here, since it has the strongest sulfate formation and ADE impact on solar radiation. The influence of ADEP in January is mainly reflected in the reduction of sulfate formation (AERO, Fig. 5a, red). This effect occurs at almost all altitudes and is greater at lower altitudes. ADED is mainly reflected in the weakening vertical transport (VTRN) of sulfate (Fig. 5b, purple) caused by a shallower PBL. Further, the weakening VTRN caused by ADED results in an increase of sulfate concentration below 500 m and decreased sulfate concentration above 500 m. The dividing point is at a similar altitude to daily max PBL height. Moreover, the dynamic path barely changes the AERO process (Fig. 5b, red). It implies that ADED affects sulfate concentration mainly by concentrating sulfate in the near-surface layer rather than changing SO_2_ concentration and sulfate formation. Compared with winter, ADED changes sulfate by promoting sulfate formation in July (Fig. 5e, red and green). ADEP on aerosol formation is negative in winter but positive in summer (Fig. 5d, red). This is mainly due to the different roles of light-absorbing and scattering aerosols in photolysis. Usually, scattering aerosol increases the optical-path length and raises the total actinic flux in the atmosphere as a whole, while absorbing aerosol decreases the actinic flux in the layer below (Dickerson et al., 1997; Herman et al., 1999). In winter, coal combustion and biomass burning, especially for residential heating, leads to high levels of light-absorbing carbon, which results in decreased actinic flux and weakened sulfate formation. Contrarily, a lower fraction of light-absorbing aerosol increases actinic flux, promoting sulfate formation in July.

**Figure 6.**
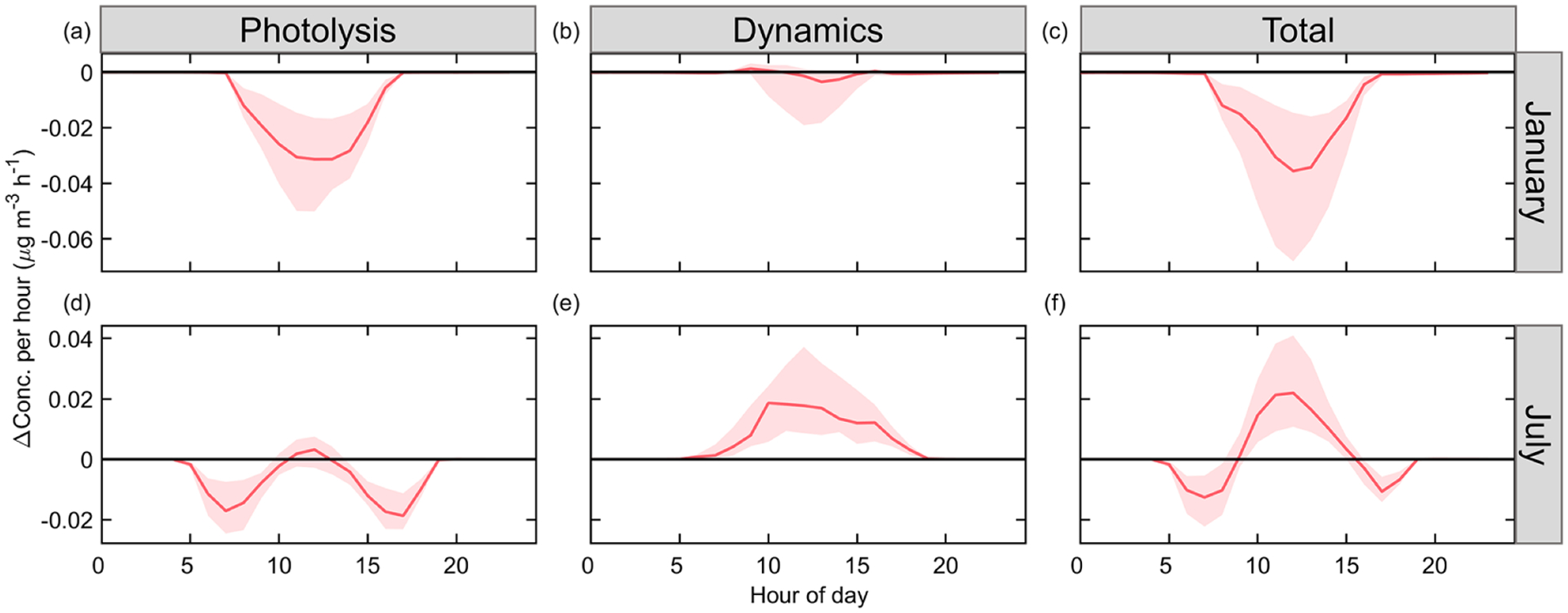
Diurnal variances of ADE impact on AERO of sulfate in January and July. The red line and shadow depict the medium value and 25th to 75th percentiles, respectively.

The results above indicate that solar radiation is the restricting factor in winter, and the formation of sulfate is sensitive to the perturbation of solar radiation. In summer, solar radiation is abundant, and sulfate formation is primarily limited by the availability of gaseous precursors. Diurnal variation of sulfate formation further verifies the above speculation. Figure 6 shows that ADEP inhibits surface sulfate formation during daytime in January, since aerosol with low SSA (single-scattering albedo) and a long optical-path length reduces the actinic flux. In July, ADEP restrains sulfate formation in the early morning and late afternoon yet slightly promotes sulfate formation at noon. Along with the strong ADED effects, sulfate formation is promoted from 10:00 to 15:00 local time in summer.

**Figure 7.**
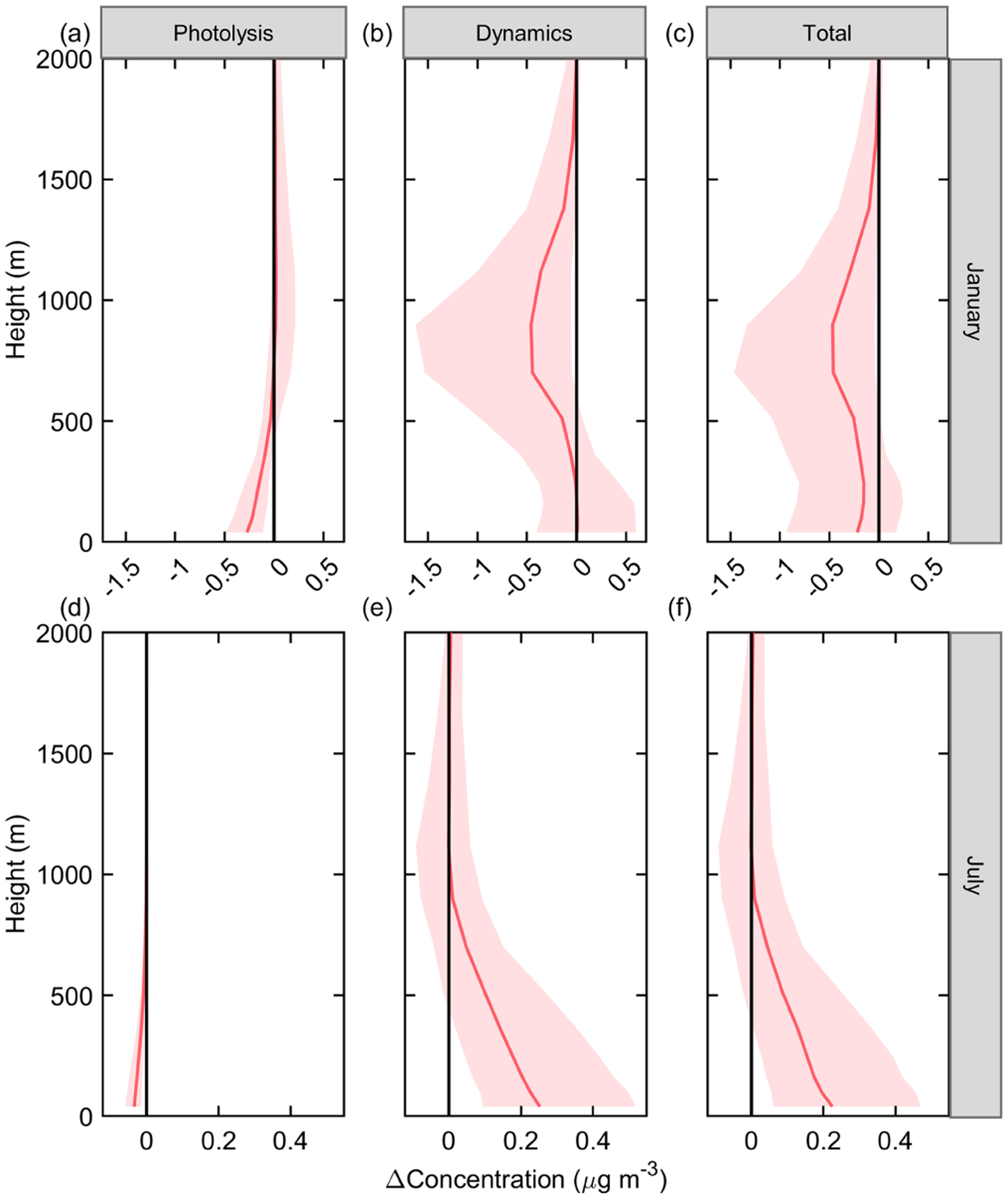
Vertical profile of nitrate concentration change to ADEs in the JJJ region at noontime in January **(a, b, c)** and July **(d, e, f)**.

**Figure 8.**
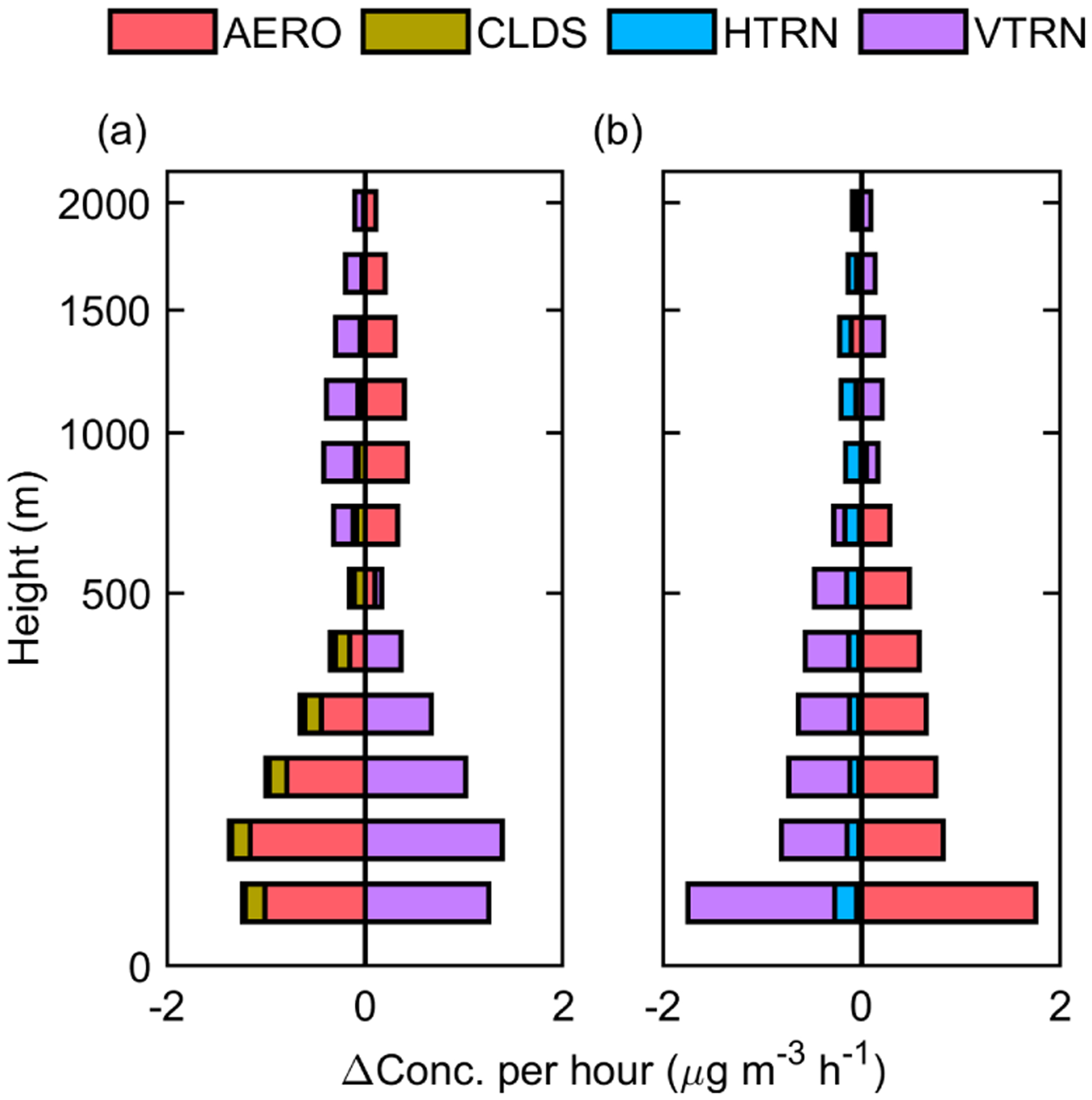
The monthly mean of vertical distribution of main process of nitrate in January **(a)** and July **(b)**.

The ADE impacts on nitrate are then investigated. The vertical profile of nitrate affected by ADEs is presented in Fig. 7. Overall, ADED has a stronger influence on nitrate concentration than ADEP in both winter and summer. ADEP slightly reduces nitrate concentration near the surface in both seasons (Fig. 7a and d). As for ADED, it generally lowers the nitrate concentration in winter (Fig. 7b), and the largest reduction occurs above the PBL (at around 900 m). During summer, ADED exhibits a promotion effect on nitrate, especially in the near-surface layers (Fig. 7e). The reason for such different impacts of ADED is caused by the opposite transport direction in January and July (Fig. 8). As shown in Fig. 8, nitrate is mainly formed at high altitude due to the lower temperature in January and is entrained to the surface with PBL development, which is also noted in previous studies (Huang et al., 2021; Curci et al., 2015). Meanwhile, the suppressed PBL reduces the upward transport of NO_*x*_ (major precursor of nitrate), resulting in weakened nitrate formation at around 900 m in winter. Conversely, the transport direction of nitrate is bottom up in July. Therefore, restrained upward transport of NO_*x*_ increases the formation of nitrate in the near-surface layer.

**Figure 9.**
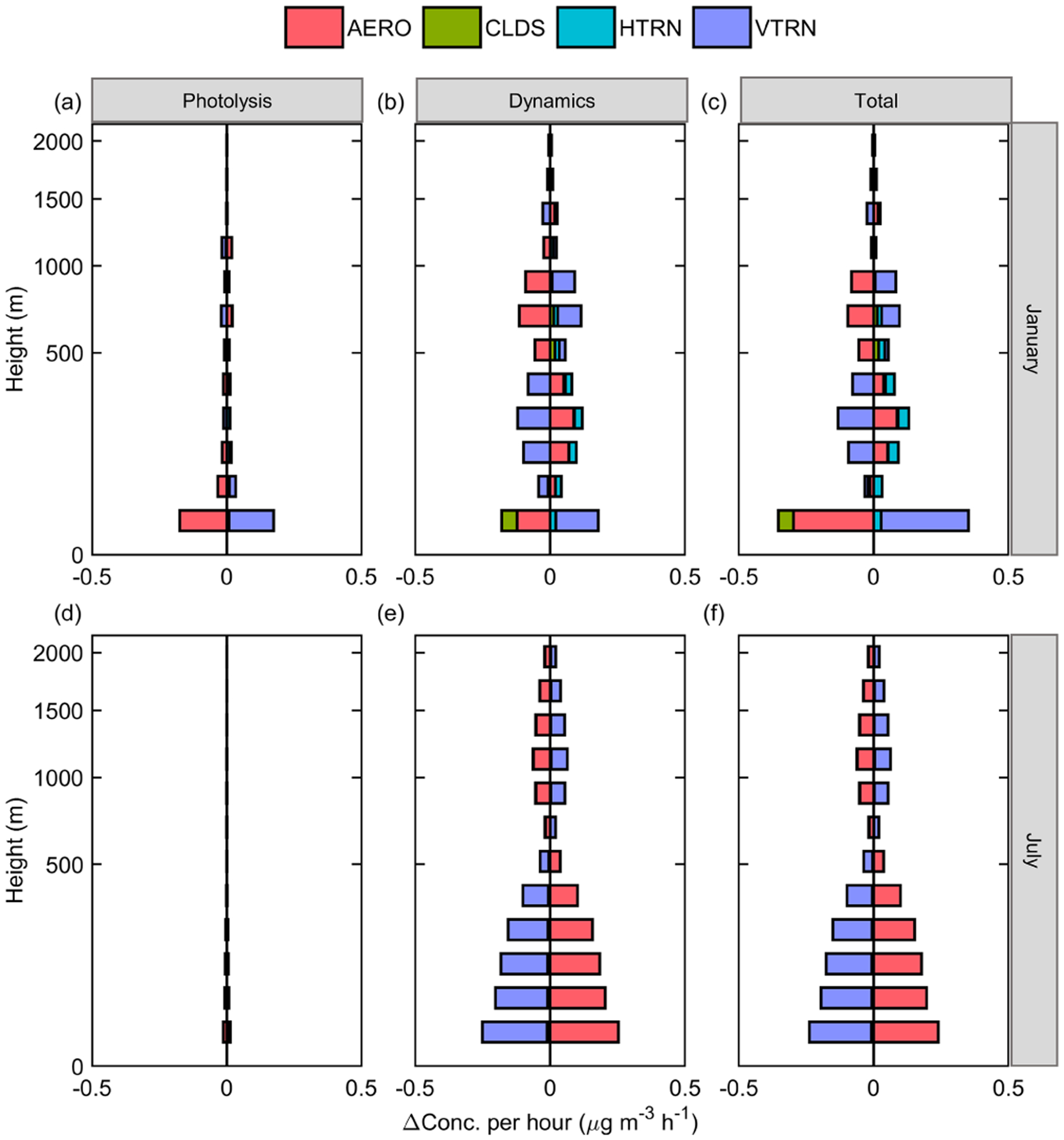
Vertical distribution of the responses of the main process of nitrate to ADEs in the Jing-Jin-Ji (JJJ) region in January **(a, b, c)** and July **(d, e, f)**.

The vertical distribution of the nitrate IPR response to ADEs is presented in Fig. 9. ADEP increases nitrate consumption (AERO, Fig. 9a, red) in the near-ground layer in January, while it barely changes the nitrate formation in July (Fig. 9d, red). In general, ADED is dominant in the upper layers in January and in all layers in July. ADED affects nitrate concentration through two major pathways, i.e., vertical transport (shown in Fig. 9b and e, purple) and precursor concentration with further impact on formation (shown in Fig. 9b and e, red). During winter, AERO is the main sink in the near-ground layers, and the transport direction is top down. Decreased nitrate formation (Fig. 9b, red) outside the PBL and a suppressed PBL result in the weakened vertical transport of nitrate (Fig. 9e, purple) and a decrease of its concentration within the PBL. In summer, AERO is the main source, and VTRN is the major sink. The main reason for increased nitrate concentration is that the accumulation of gaseous precursors in the PBL enhances nitrate formation (Fig. 9e, red). This effect further increases the absolute amount of nitrate transportation.

## 4 Conclusions

In addition to directly deteriorating air quality, aerosol diminishes solar radiation due to light scattering and absorption, thereby influencing regional meteorology and further modulating air quality. The impact of ADEs on secondary aerosol is more complicated than primary aerosol. This study quantified the impacts of ADEs on secondary inorganic aerosol using the two-way online coupled meteorology and atmospheric chemistry model (WRF–CMAQ) with integrated process analysis. The main pathways through which ADEs affect aerosol concentrations were examined. The key conclusions are the following. (1) ADEs reduce solar radiation and decreases PBL height, concentrating aerosol in the near-ground layers. In this analysis, ADEs improved the model performance for simulating PM_2.5_ and its components. (2) ADEs through the photolysis pathway inhibit sulfate formation during winter in the JJJ region and promote sulfate formation in July. The differences are attributed to the alteration of effective actinic flux affected by aerosol optical depth (AOD), solar zenith angle, and SSA. (3) ADEs through the dynamics pathway act as an equally or even more important route compared with the photolysis pathway in affecting secondary aerosol concentration in both summer and winter. (4) ADEs through dynamics traps formed sulfate within the PBL, which increases sulfate concentration in winter. Meanwhile, the impact of ADEs through dynamics is mainly reflected in the increase of gaseous-precursor concentrations within the PBL, which enhances secondary aerosol formation in summer. (5) Reduced upward transport of precursors restrains the formation of nitrate at high altitude and eventually lowers the nitrate concentration within the PBL in winter, while such weakened vertical transport of precursors increases nitrate concentration within the PBL in summer, since nitrate is mainly formed near the surface ground.

## Supplementary Material

Supplement1

## Data Availability

Model outputs are available upon request from the corresponding author.
