# Supplementary material for: The pathway of impacts of aerosol direct effects on secondary inorganic aerosol formation
Source: Atmos Chem Phys. Author manuscript; Available in PMC 2023 Apr 20. (PMC9413026; doi:10.5194/acp-22-5147-2022)
Supplement: Supplement1 [file NIHMS1826718-supplement-Supplement1.pdf]

Supplement of Atmos. Chem. Phys., 22, 5147–5156, 2022
https://doi.org/10.5194/acp-22-5147-2022-supplement
© Author(s) 2022. CC BY 4.0 License.

Supplement of

The pathway of impacts of aerosol direct effects on secondary inorganic aerosol formation

Jiandong Wang et al.

Correspondence to: Jia Xing (xingjia@mail.tsinghua.edu.cn)

The copyright of individual parts of the supplement might differ from the article licence.

1 Model evaluation

The simulated concentrations of surface SO_2, NO_2 and $PM_{2.5}$ in SimNF (no aerosol feedbacks) and SimSF (which aerosol feedbacks) are compared with observed data in Figure S2. In January, high SO_2 concentrations are shown in JJJ, YRD, HUZ, and SCH. In general, simulated SO_2 concentration is underestimated in JJJ. The low-bias is getting larger under high $PM_{2.5}$ level, shown in Figure S2. JJJ region is with highest observed SO_2 value up to 500 μg m^{-3}. Meanwhile, SO_2 concentration is overestimated in PRD, HUZ, and SCH. The simulated SO_2 match pretty well with the observation in YRD. ADE increases SO_2 concentration in most regions, except eastern Henan and middle Shandong where is the downwind area of polluted regions. The enhanced atmospheric stability reduced the ventilation condition resulting in an increased polluted level at source area but decreased polluted level at downwind area. The increase of SO_2 is up to 56 μg/m^3 in the polluted regions. In July, high SO_2 concentrations are still shown in JJJ, YRD, PRD, HUZ, and SCH, but much lower than in January. SO_2 concentration is lower than 50 μg/m^3 in most cities, except Handan (south of JJJ). Model generally overestimates SO_2 concentration in most regions. ADE enhances SO_2 concentration in part of JJJ, YRD, and SCH. But SO_2 is decreased due to ADE in PRD. NO_2 also exhibits higher concentration in January and lower concentration in July. High NO_2 is usually located at large cities. In January, high NO_2 is shown in Northeast China, JJJ, HUZ, and YRD. The cities in south part of JJJ, i.e., Beijing, Tangshan, Baoding, Shijiazhuang, Xingtai, and Handan are the most polluted cities where monthly averaged NO_2 concentrations exceed China air quality standard of daily average NO_2 concentration (i.e., 80 μg/m^3). In general, the model slightly underestimates NO_2 for most regions. ADE enhances NO_2 concentration by over 19.7 μg/m^3 in JJJ, YRD, HUZ, and SCH, which improves the model performance. In July, the NO_2 concentration is much lower than in January. The model also underestimated NO_2 concentration. $PM_{2.5}$ concentrations in January exceed 160 μg/m^3 in all 5 regions. The model generally underestimates $PM_{2.5}$ concentrations in almost all regions. ADE enhances monthly averaged $PM_{2.5}$ concentrations by over 2 μg/m^3 in most area of East China. The maximum increase reached 35.8 μg/m^3. Compared to January, $PM_{2.5}$ concentrations in July are much lower and mostly high concentrations are located in JJJ and part of SCH. Simulated $PM_{2.5}$ concentrations match well with the observed data.

2 Impact of ADE on oxidants

To further investigate the impacts of ADE on atmospheric chemistry, we examined the changes in production rates of new reacted OH, shown in Fig S5. The modification of atmospheric oxidants by ADE also shows solar radiation control in January and gaseous precursor control in July. In January, ADEP is the dominant process to impact atmospheric oxidation. It leads to a decrease of oxidants in the layer below 1 km and an increase in oxidants above it. ADED slightly raises oxidation near ground and exhibits little impact on layers above 500 m. In July, both dynamic and photolysis pathways are important. ADEP increases atmospheric oxidants in all layers. The height with strongest effect is about 600 m. ADED amplifies near-surface atmospheric oxidants but reduces atmospheric oxidants above 600 m.

3 Impact of ADEP on sulfate

The influence of changes in the photolysis pathway on aerosol formation is negative in winter and positive in summer. This is mainly due to the different effects of light absorption and scattering on aerosols and surface albedo. Usually, scattering aerosol increases the effective optical path length and raises the total actinic flux in the atmosphere as a whole, while absorbing aerosol decreases the actinic flux in the layer below, compared with an aerosol-free scenario (Dickerson et al., 1997;Herman et al., 1999). The influence of aerosol on the photochemical reactions also varies with single scattering albedo (SSA). A low SSA value (strong absorption) tends to inhibit the photochemical reaction, while a high SSA tends to promote the photochemical reaction. Moreover, such impact varies with altitude and aerosol loading. Forward scattering increases actinic flux of the layer

below, given that the diffuse light increases the effective optical path length. Backward scattering increases the actinic flux of the layer above the aerosol but decreases the actinic flux below the aerosol layer. Thus, the ground-level actinic flux will depend on aerosol loading and vertical distribution. The factors impacting actinic flux include but are not limited to single scattering albedo, aerosol loading (aerosol optical depth, τ) and solar zenith angle (θ). Higher effective optical depths (τ /cos θ, a variable to represent aerosol loading) attenuate direct solar radiation. Thus, this impact will be more significant at high θ (Dickerson et al., 1997;He and Carmichael, 1999) and high τ. In January, the average AOD reached 2.5, much higher than the annual average level (Bi et al., 2014). Coal combustion and biomass burning, especially for residential heating, leads to high levels of black carbon, which results in low SSA. High aerosol loading, low SSA, and low solar zenith angle together lead to decreased actinic flux in near-ground layers, due to ADE. Conversely, low aerosol loading, high SSA, and high solar zenith angle together lead to increased actinic flux in near-ground layers in July.

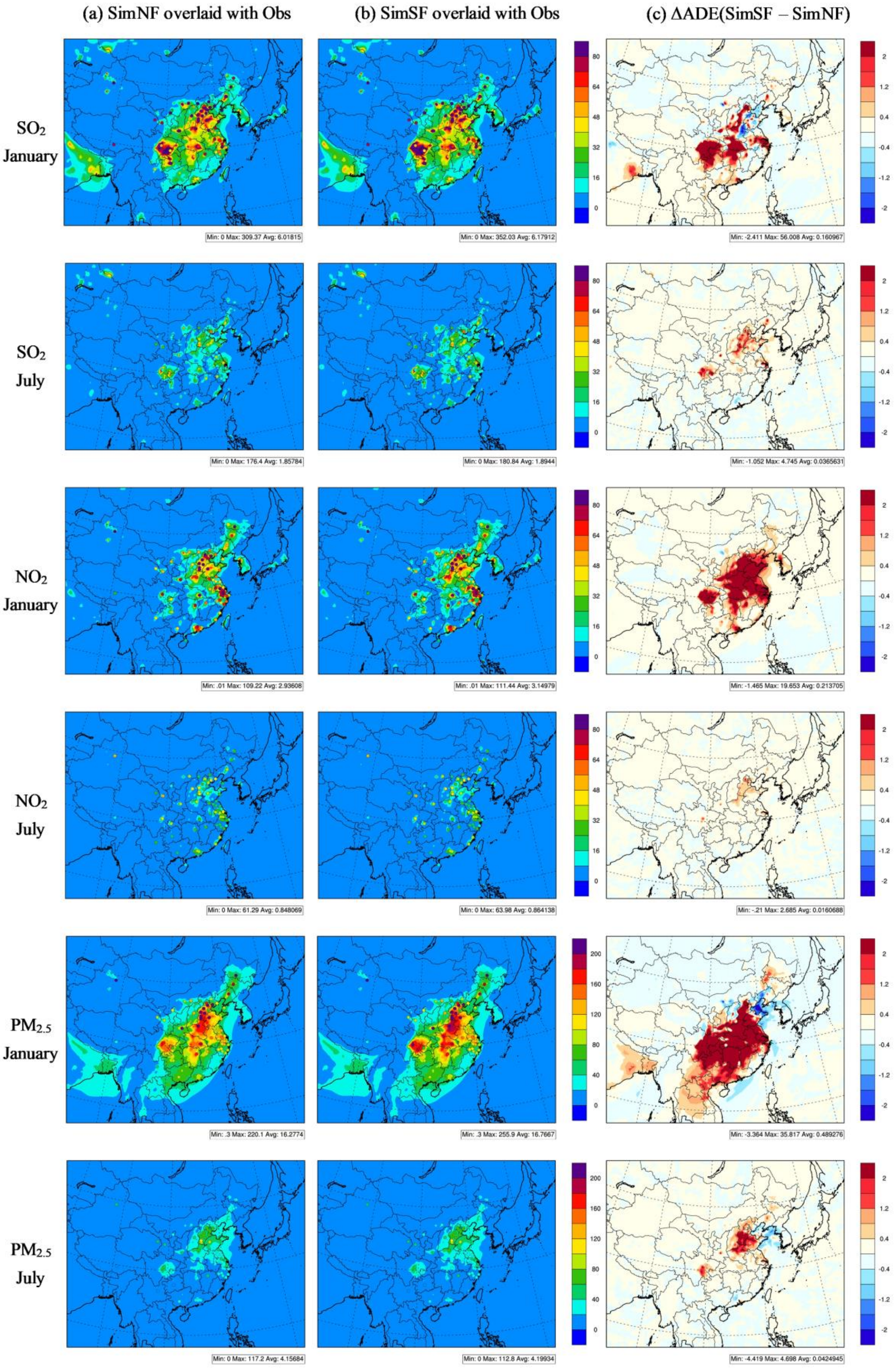

Figure S1. Observed and simulated SO_2, NO_2 and $PM_{2.5}$ and their responses to ADE (monthly mean, μg m-

^{3})

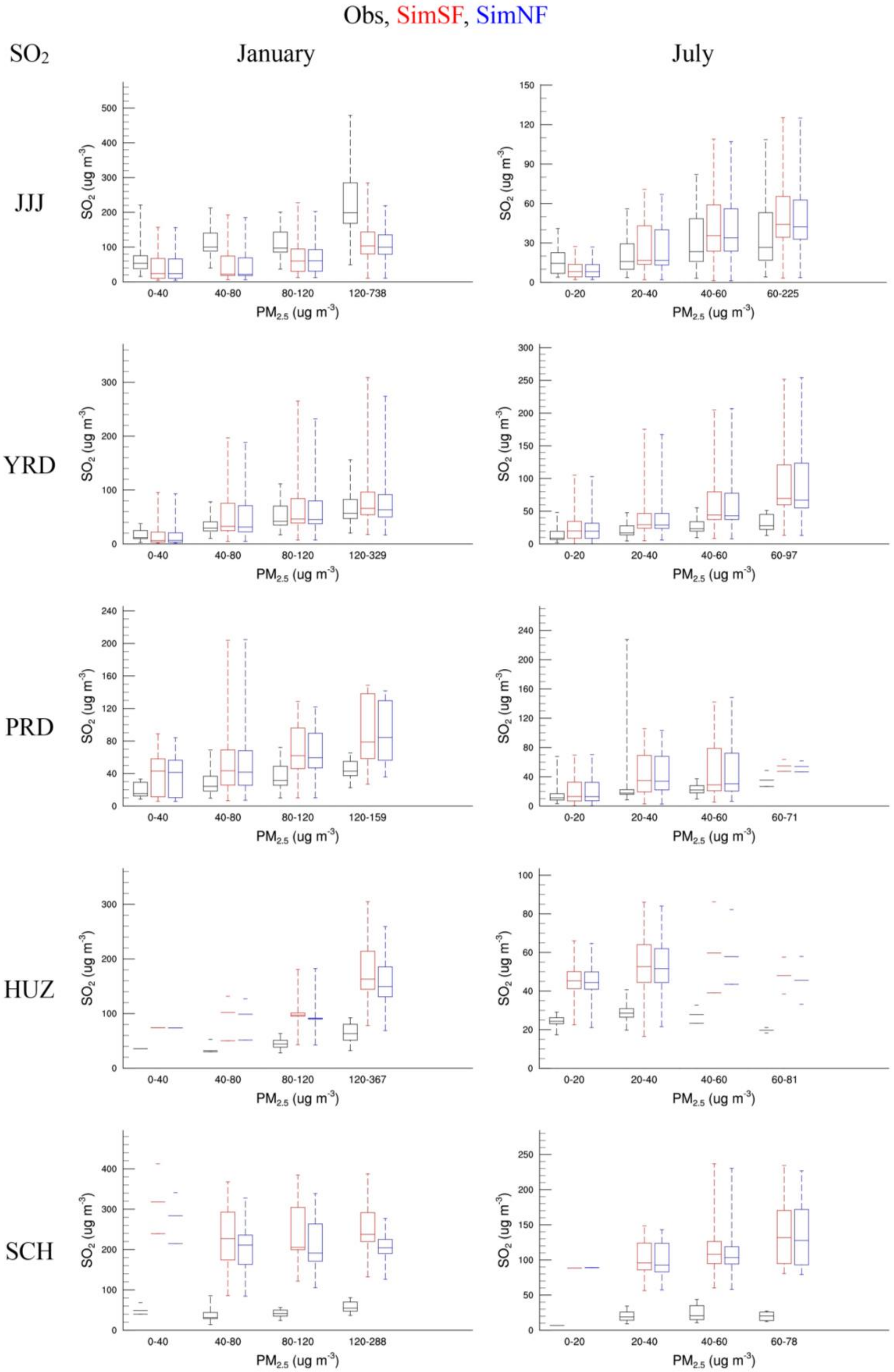

Figure S2 Observed and simulated surface SO_2 concentration against $PM_{2.5}$ concentration (monthly mean, μg m^{-3})

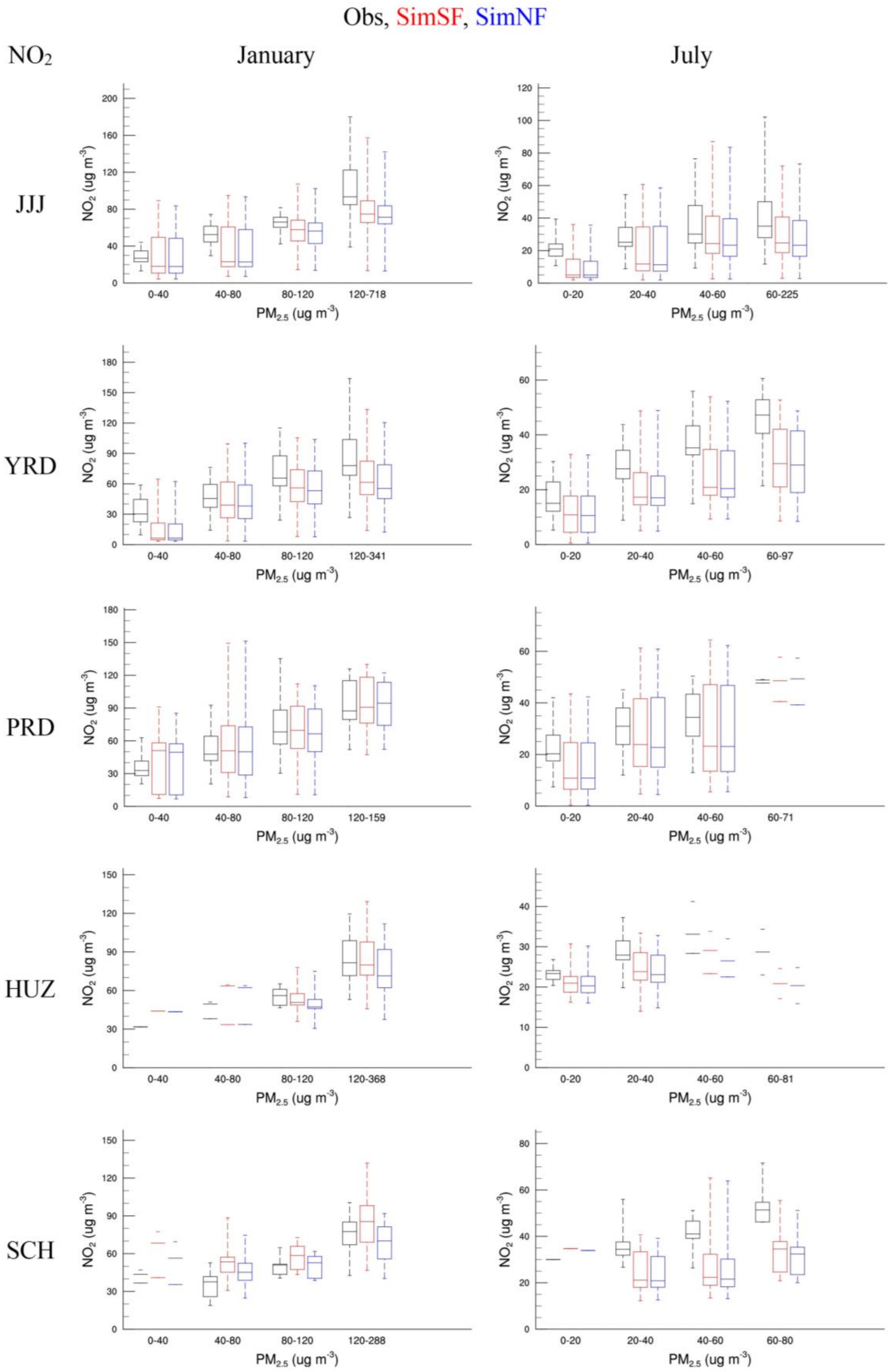

Figure S3 Observed and simulated surface NO_2 concentration against $PM_{2.5}$ concentration (monthly mean, μg m^{-3})

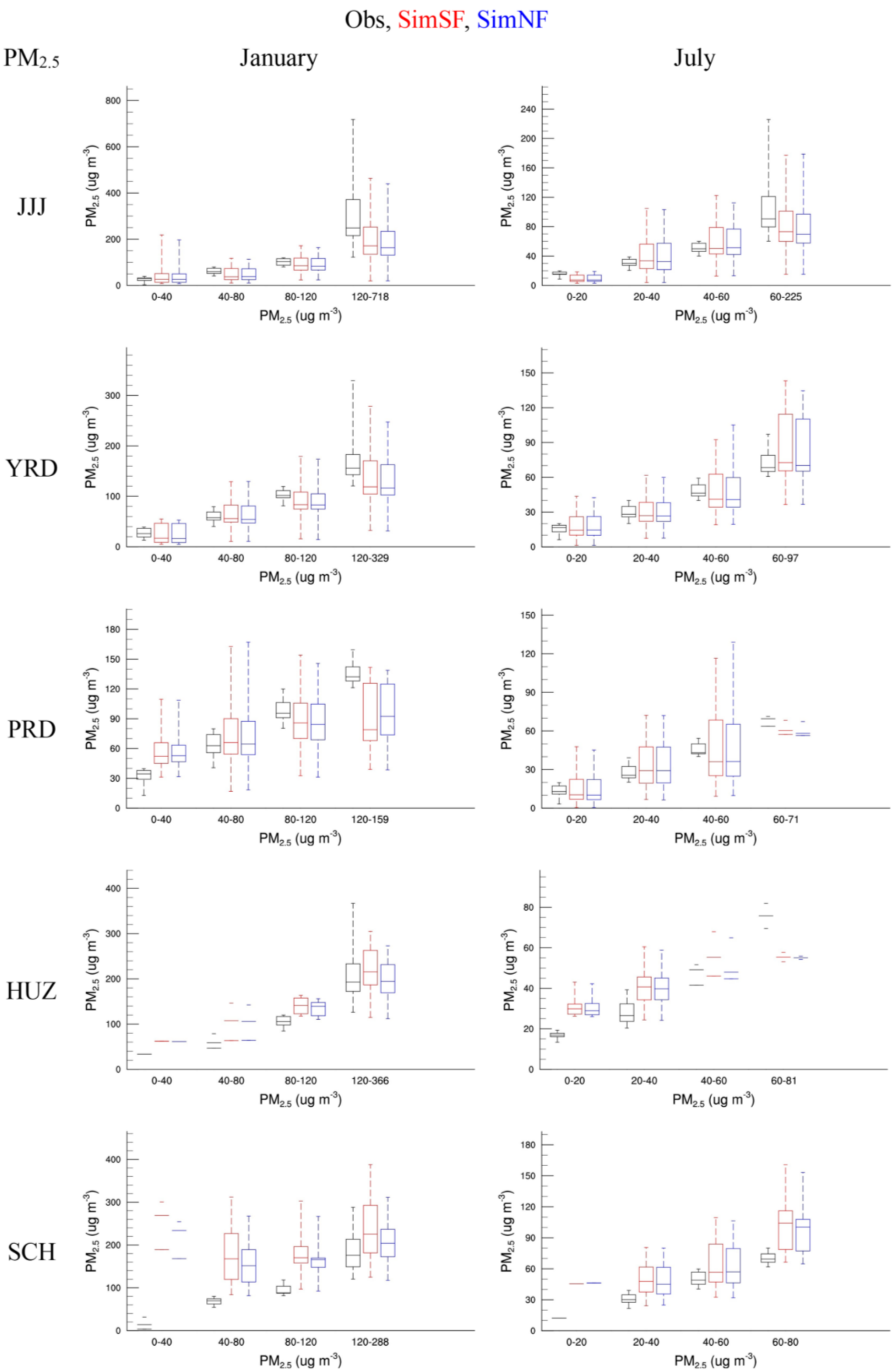

Figure S4 Observed and simulated surface $PM_{2.5}$ concentration (monthly mean, $\mu g\ m^{-3}$)

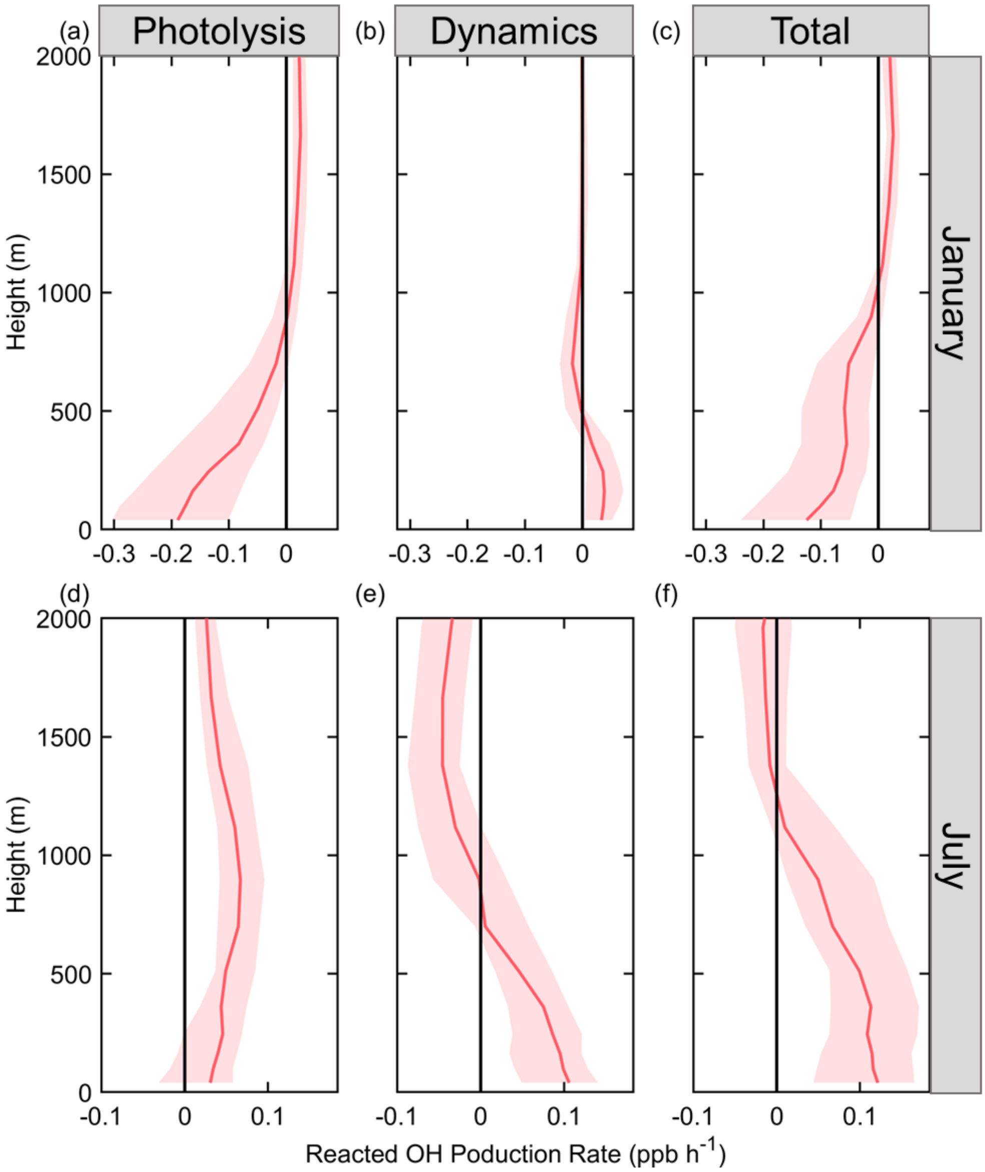

Figure S5: Vertical distribution of ADE impact on mean reacted oxidation production. The red line and shadow show the medium value and 25th to 75th percentiles, respectively.
